# Current knowledge and challenges associated with targeted delivery of neurotrophic factors into the central nervous system: focus on available approaches

**DOI:** 10.1186/s13578-021-00694-2

**Published:** 2021-10-12

**Authors:** Gozal Bahlakeh, Reza Rahbarghazi, Daruosh Mohammadnejad, Ali Abedelahi, Mohammad Karimipour

**Affiliations:** 1 Neurosciences Research Center (NSRC), Tabriz University of Medical Sciences, Tabriz, Iran; 2 Department of Anatomical Sciences, Faculty of Medicine, Tabriz University of Medical Sciences, Tabriz, Iran; 3 Stem Cell Research Center, Tabriz University of Medical Sciences, Tabriz, Iran; 4 Department of Applied Cell Sciences, Faculty of Advanced Medical Sciences, Tabriz University of Medical Sciences, Tabriz, Iran

**Keywords:** Neurotrophic factors, Delivery system, Neuroregeneration, Administration routes

## Abstract

During the last decades, numerous basic and clinical studies have been conducted to assess the delivery efficiency of therapeutic agents into the brain and spinal cord parenchyma using several administration routes. Among conventional and in-progress administrative routes, the eligibility of stem cells, viral vectors, and biomaterial systems have been shown in the delivery of NTFs. Despite these manifold advances, the close association between the delivery system and regeneration outcome remains unclear. Herein, we aimed to discuss recent progress in the delivery of these factors and the pros and cons related to each modality.

## Introduction

**Fig. 1:**
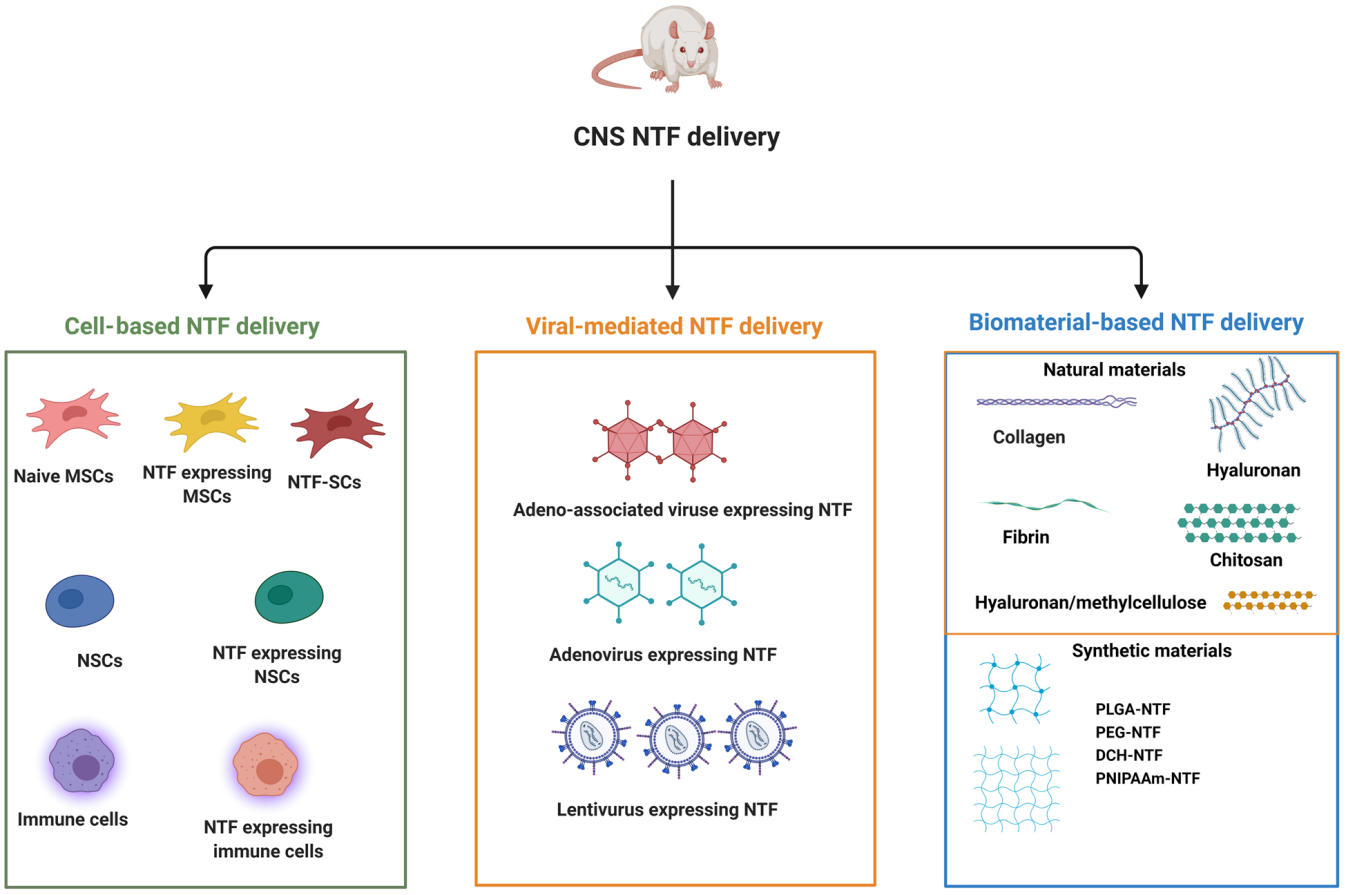
Different stem cells and cells can be utilized as a vehicle for neurotrophic factor (NTF) delivery. Mesenchymal stem cells (MSCs) can be utilized as three different cells: the naïve MSCs can secrete NTF when transplanted in the CNS of rodents. The MSCs can be engineered to overexpress a kind of NTF or can overexpress a variety of NTF when is differentiated to Neurotrophic factor secreting cells (NTF-SCs). Neural stem cells (NSCs) can be transplanted as naïve NSCs or NTF overexpressed NSCs for NTF delivery. The immune cells like macrophages can be engineered to overexpress NTF. Different viruses were utilized as vehicles for NTF delivery. AdV; Adenovirus, AAV; Adeno-associated virus, LV; Lentivirus. Different biomaterials, both natural and synthetic ones were utilized for NTF delivery into the CNS. HAMC; hyaluronan/methylcellulose, PEG; poly (ethylene glycol), PNIPAAm; Poly(N-isopropylacrylamide), DCH; Amphiphilic diblock copolypeptide hydrogel, HyStem; HyStem®-C hydrogel, PLGA; Poly (lactic-co-glycolic acid)

In contrast to different tissues, CNS has low-rate regenerative capacity following acute and chronic neurological disorders. This feature of the CNS has been led to the development of novel, supportive and restorative strategies in regenerative medicine [1]. During the developmental and adult periods, NTFs per se support optimum milieu to regulate cellular bioactivity and tissue organization via engaging both cellular and molecular mechanisms [2]. Upon the onset of neurological disorders, CNS produces endogenous NTFs in response to external and internal insults to restore structural and functional plasticity of injured neurons. Unfortunately, these compensatory responses are not often sufficient and effective in a prolonged period. In addition to the reduction of endogenous NTF production, loss of equilibrium ratio can also happen in favor of specific factors, leading to neurodegenerative disorders including AD, PD, Huntington, and ALS, etc. It has been indicated that timely right dose introduction of such types of NTFs can impede the worsening of pathological conditions. Besides, pieces of evidence have shown that peripheral administration of NGF causes pain and weight loss [3, 4]. Commensurate with these descriptions, development and introduction de novo effective administration approaches are subject of debate. In this regard, timely and controllable delivery of NTF cocktails should not be neglected in developing approaches. This review will describe several available delivery systems into the CNS by focusing on different animal models. The discovery and development of appropriate NTF delivery system (s) will enable us to manage the occurrence and progression of the neurodegenerative disease using single and combined growth factor therapies (Fig. 1).

## Types and importance of NTFs in CNS

**Fig. 2:**
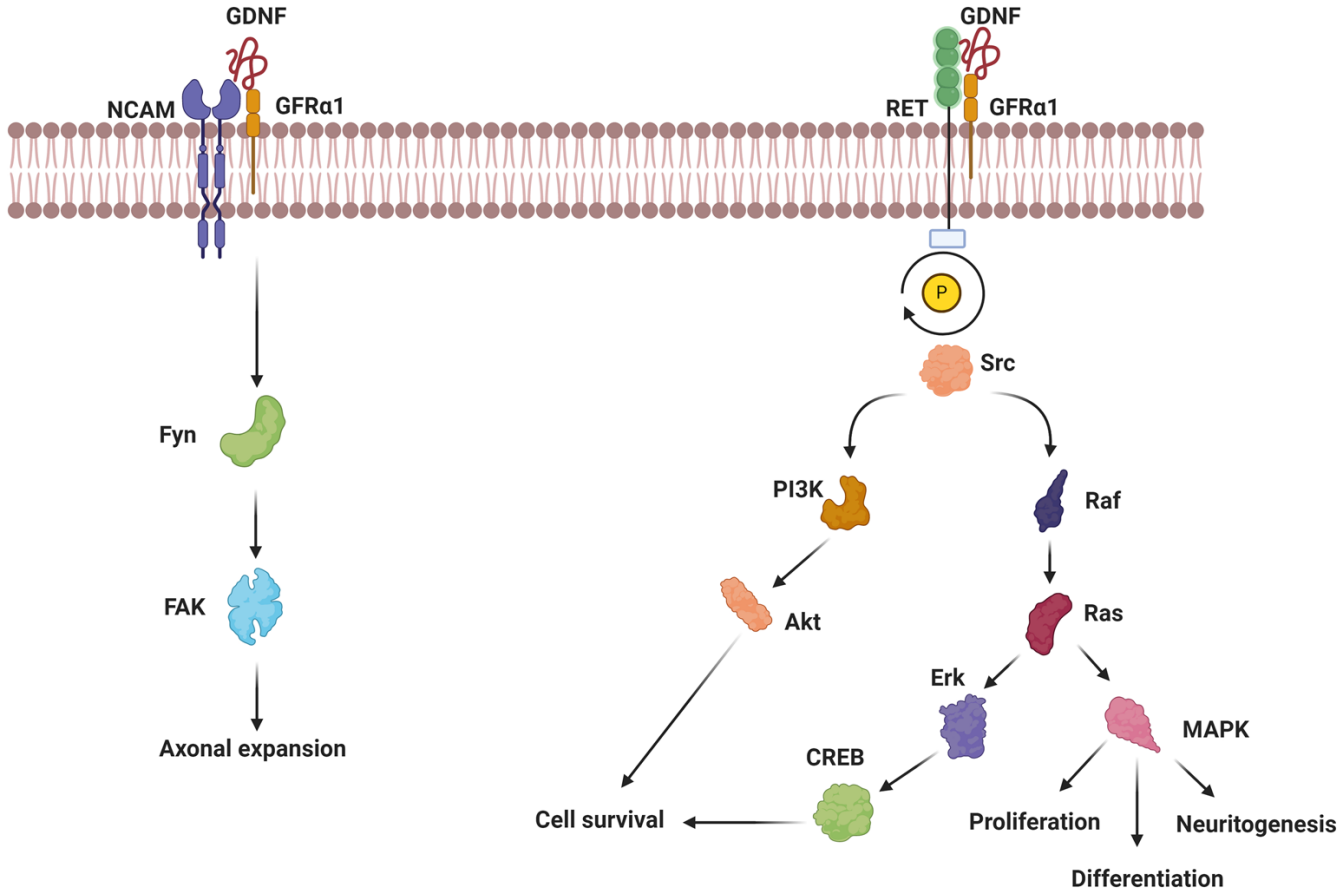
GDNF/RET signaling pathway. The GDNF/GFRα1 complex triggers the intracellular signaling pathway through phosphorylation of the RET tyrosine kinase receptor. The RET in turn activates Src family tyrosine kinase. The activation of Src triggers the Raf/Ras/MAPK cascade leading to proliferation, differentiation, and neuritogenesis. The Raf/Ras/ERK/CREB pathway in collaboration with PI3K/AKT axis induces cell survival in the brain context. In the GDNF/NCAM pathway, the GDNF/GFRα1 complex recruits the NCAM which in subset activates the Fyn/FAK complex. These changes contribute to axonal expansion

Generally, neuroprotection refers to structural and functional preservation of the neurons and glial cells inside the CNS under degenerative and neuro-inflammatory conditions [5]. NTFs are a group of diffusible peptides or small proteins that are essential for neural survival and growth in the CNS and PNS [6, 7]. During the occurrence of pathological conditions, the normal physiological role of NTFs can be altered. As matter of fact, the imbalance of NTFs, either transportation rate or basal levels, can result in neural cells death and degeneration [3]. From the past to the present, near 50 types of NTFs have been identified. Further molecular and biological studies have shown that NTFs compass several families as follows; GDNF, Neurotrophin, Neuropoietin, and CDNF/MANF families [8] (Fig. 2 and Table 1).

**Table1:** A summary of NTFs delivery vectors in CNS and their possible improvement pathways

Vectors/Routes	Neurotrophic factors		Disease model	Related pathways	Possible consequence of neurotrophic factor
Cell/stem cell	Mesenchymal stem cells	IGF-1	SCI rats	Axonal Extension/survival	Extended the axons and improved the corticospinal motor neurons
		BDNF	MS mice	Inflammation	Raised BDNF and GAD67and the L-4,-10,-1, diminished TNF-α and IFN-γ and improved re-myelination
		GDNF	PD rats	Dopamine pathway	Enhanced sprouting of dopaminergic terminals
		NT-3	SCI rats	Axonal extension/survival	Induced motor function, axonal regrowth and survival
		NGF BDNF NT-3	TBI rats	Apoptosis	Enhanced p-Akt and decreased caspase-3
		BDNF	PD rats		Enhanced TH positive cell number, PCNA expression and motor function
		CNTF	SCI rats		Improved BBB test performance
		BDNF	HD mice	Neurogenesis	Diminished striatum atrophy and anxiety and raised neurogenesis and life span
	Neural stem cells	BDNF	AD mice	Synaptic pathways	Improved synaptic density and behavioral function
		GDNF	ALS rats		Caused differentiation in astrocytes
	Neurotrophic factor secreting cells	BDNF GDNF NGF	HD rats		Could migrate to lesion site
			PD rats	Survival pathways	Enhanced survival of DA neurons and improved motor and behavioral functions
			PD rats	Regeneration	Upregulated dopamine level and regenerated the network of DA nerve end in striatum
			MS mice		Decreased the disease symptom and enhanced life span
			HD rats	Neuroprotection	Improved behavioral function
			SCI rats	Remyelination/survival	Improved myelin and raised the number of oligodendrocyte
			MS rats		Enhanced MBP and Olig2 proteins expression
	Immune cells	GDNF	PD rats	Axonal regeneration/survival	Improved open field activity, regenerated axons and preserved TH positive neurons
		NTN	PD rats	Synaptic pathway/survival	Preserved TH positive neurons, improved synapses and behavioral function
		GDNF	PD mice		Could cross the BBB and delivered GDNF to the DA neurons
		GDNF	PD mice	Inflammation/proliferation	Enhanced motor function and DA neuron number and reduced α-synuclein and inflammation
		GDNF	PD mice	Dopamine pathway	Raised TH positive neurons number and motor/non motor function
	C2C12 cells	CNTF	AD mice	Synaptic pathway/survival	Restored synapses and survival signaling and cognitive function
	hUCBCs	VEGF GDNF	ALS mice		Improved behavioral performance and increased the mean life-span
	HEK293	GDNF	PD mice		Enhanced TH positive neurons and motor function
Virus	Adeno-associated virus	IGF-I	ALS rats		Improved motor function in male rats
		BDNF	SCI rats	Apoptosis	decreased caspase-3 and upregulated NG2 expressing cell number
		EPO	PD rats		Bettered DA neurons population and motor function
		GDNF	Healthy rats		Rapamycin regulated GDNF releasing
		IGF-1	SMA mice	Apoptosis	Restrained apoptosis
		BDNF	SCI rats	Axonal regeneration	Regenerated axons but worsened motor function and caused spasticital symptoms
		BDNF	AD mice	Synaptic pathway/survival	Improved behavioral function, neuronal survival and synapses
	Lentivirus	BDNF	SCI rats	Axonal expansion	Elongated axons in neurons
		BDNF NT-3	SCI rats	Axonal regeneration	Regenerated and remyelinated axons
		NT-3	SCI rat		Improved neurons, locomotor function and diminished the astrocyte level
		NT-3 NT-3/SHH	SCI mice	Axonal regeneration	Raised axonal regeneration, remyelination and enhanced the number of glial cells
		GDNF	AD mice		Enhanced learning and memory and BDNF level but decreased cognition
Biomaterial	Microspheres	GDNF	PD rats		Ameliorated rotational behavior and extended TH positive fibers
	Chitosan	NGF	Healthy rats		Upgraded bioavailability of NGF up to 14 fold
	PNIPAAm-g-PEG PNIPAAm-g-MC	BDNF	SCI rats	Axonal regeneration	Enhanced axonal regeneration
	HAMC hydrogel	EPO	Stroke mice	Neurogenesis, inflammation, apoptosis	Enhanced neurogenesis, mediated inflammation and diminished the apoptosis
		BDNF	SCI rat	Survival pathway/inflammation	Progressed neuronal survival and decreased the expression of pro-inflammatory cytokines
	DCH	NGF	Healthy mice	Cholinergic system	Caused hypertrophy of cholinergic neurons
	Gelatin nanoparticles	bFGF	PD rats	Dopamine pathway	Improvement of DA function in synapses
	Collagen hydrogel	MSCs GDNF	Healthy rats	Survival pathway	Moderated neuroglia activation, improved cell survival and GDNF secretion
	collagen conduits	NT-3	Healthy rats	Axonal extension	Improved axonal extension
	Hyaluronan hydrogel	NT-3	SCI rats	Axonal regeneration	Regenerated and enlarged the axons, did not induce the astroglial response and improve motor function
		BDNF	Stroke mice	Axonal regeneration/survival	Regenerated axons and caused migration and survival of immature neurons
	Fibrin	NT-3	SCI rats	Synaptic pathway	Advanced neuronal fiber density and diminished glial scar
		NT3 PDGF with ESNPCs	SCI rat	Survival pathway	Enhanced ESNPC derived mature neurons and survival of ESNPC in the lesion site
	RDP	BDNF	Healthy mice	Survival	Reduced infarct volume and neural loss
	nano-particle polyion complex with PEG/PGA copolymers	BDNF	Stroke mice		upgraded memory and cognitive function and sustained myelin base proteins
	PLGA/ nanoparticles/ poloxamer 188 (PX) coated	BDNF	TBI mice		Restored cognition and neurological loss
	HyStem®-C hydrogel	BDNF	Stroke rats		Improved sensorimotor function tests, diminished infarct volume and glial markers
	PLGA/GO electrospun nanofibers	IGF-1 BDNF	SCI rats		Increased the lesion site population of neurons, locomotor function and moderated the formation of cavity
Ultrasound& MBs		BDNF	Healthy mice		Enhanced BDNF concentration in target site
		GDNF	Healthy rats		Could cross through BBB and were delivered locally in an non-invasive way
		BDNF GDNF NTN	Healthy mice		Started molecular signaling of hippocampal pyramidal neurons inside the nucleus
		NTN	Healthy mice		Upregulated the NTN levels in caudoputamen and substantia nigra, began the signaling pathway
		GDNF	PD rats		Improved motor and behavioral functions
Pump		BDNF	HD mice		Mediated function and motor coordination, lingered life time and downregulated the microglial reaction
		CNTF	MS rats	Inflammation	Restricted inflammation, diminished demyelination, axonal deficit and neuronal death
		NGF	HD mice	Cholinergic system, neurogenesis	Upregulated ChAT and VAChT levels, elevated neurogenesis
		BDNF	SCA1 mice	Synaptic pathway	Reduced motor loss and synaptic deficit of Purkinje neurons
Intranasal		BDNF, NT-4, CNTF and EPO	Healthy rats	survival pathway	Upregulated the NTF concentrations and initiated cell survival pathway
		NGF	stroke rats	survival pathway, maturation	Improved neural cell survival and maturation
		bFGF spray	AD rats	Cholinergic system	Enhanced ChAT and acetylcholinesterase activity and decreased hippocampal neuronal degeneration
		NGF	TBI rats	Amyloid pathway	Reduced Aβ1-42 deposits and recovered the motor and behavioral function
		GDNF	PD rats		Increased TH positive neurons and DA cells and several constant dose of NTF were more efficient

*NTF* neurotrophic factor, *GDNF* Glial cell-derived neurotrophic factor, *NGF* Nerve growth factor, bFGF; Basic fibroblast growth factor, *BDNF* Brain derived neurotrophic factor, *NT-4* Neurotrophin-4, *CNTF* Ciliary neurotrophic factor, *EPO* Erythropoietin, *NTN* Neurturin, *IGF-1* Insulin-like growth factor-1, *NT-3* Neurotrophin-3, *PDGF* Platelet-derived growth factor, *RDP* Rabies virus glycoprotein, *DCH* Diblock polypeptide hydrogels, *SHH* Sonic hedgehog, *MSCs* Mesenchymal stem cells, *ESNPCs* Embryonic stem cell-derived neural progenitor cell, *SCI* Spinal cord injury, *MS* Multiple sclerosis, *PD* Parkinson’s disease, *TBI* Traumatic brain injury, *HD* Huntington’s disease, *AD* Alzheimer’s disease, *ALS* Amyotrophic lateral sclerosis, *SMA* Spinal muscular atrophy, *SCA1* Spinocerebellar ataxia type 1, *GAD67* Glutamic acid decarboxylase 67, *TNF-α* Tumor necrosis factor alpha, *IFN*-*γ* *Interferon gamma,* *pAkt* protein kinase B, *TH* Tyrosine hydroxylase, *PCNA* Proliferating cell nuclear antigen, *DA* Dopaminergic, *MBP* Myelin basic protein, *BBB test* Basso-Beattie-Bresnahan test, *BBB* Blood- brain barrier, *NG2* Precursor of oligodendrocyte lineage, *ChAT* Choline acetyltransferase, *VAChT* Acetylcholine transporter, *Aβ* Amyloid beta, *PLGA*/*GO* Poly lactic-co-glycolic acid/Graphene oxide *PEG/PGA* Poly (glutamic acid)-poly (ethylene glycol), *C2C12* cells Myoblast line, *hUCBCs* Human umbilical cord blood cells, *HEK293* Human embryonic kidney 293 cell line

The family of GDNF includes the GDNF, artemin, NTN, and persephin [9]. The two most important subsets of this family, including GDNF and neurturin, can regulate the survival and maintenance of midbrain DA neurons [10]. All of these family members exert their regulatory functions via the GDNF family receptor alpha (GFRα1-4) and subsequent proto-oncogene RET, a receptor tyrosine kinase receptor (Fig. 2) [8, 11]. Based on molecular investigations, the cerebellum, pons, and thalamus express the RET as the main GDNF receptor [9, 12]. In addition to RET, the GDNF/GFRα1 complex can induce axonal expansion through NCAM, syndecan-3, integrins (e.g. integrin αV and β1), or N-cadherin [12]. In the RET axis, the GFRα/GDNF complex promotes the dimerization and phosphorylation of RET, leading to the activation of several downstream effectors [13]. RET activates Src family-dependent tyrosine kinase, which in turn triggers PI3k/Akt and Raf/Ras/Erk/MAP/CREB axes. It is thought that these effectors are associated with cell survival, proliferation, neuritogenesis, differentiation, and synaptic plasticity (Fig. 2) [12, 14].

**Fig. 3:**
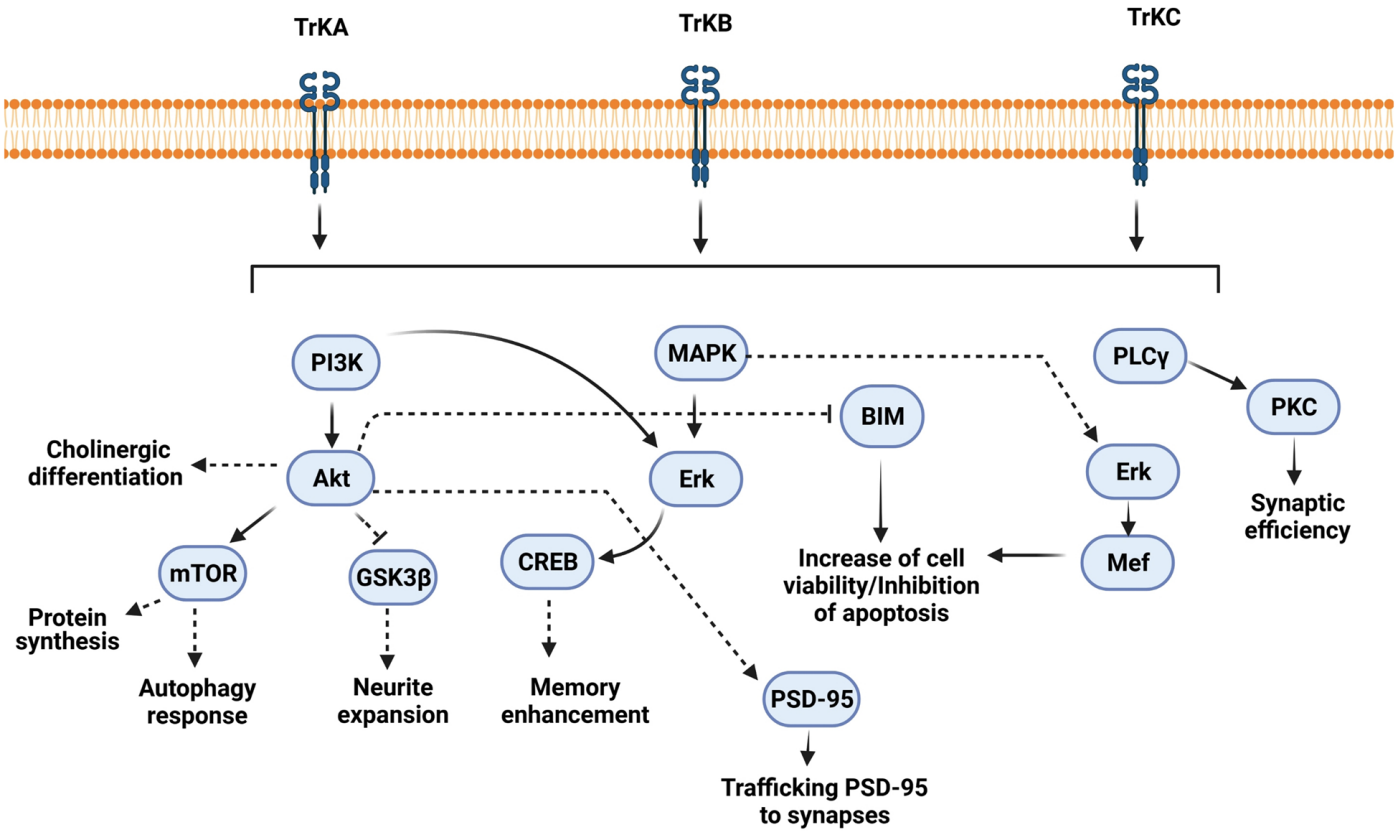
NGF and BDNF signaling pathways inside neuronal cells. After NGF binding to the TrkA receptor, several signaling pathways and down-stream effectors are activated. In this cascade, the activation of the PI3K/AKT complex regulates cholinergic differentiation. Further, the promotion of the PI3K/AKT/mTOR axis inhibits the autophagic response. In parallel, the activation of the PI3K/AKT/GSK3 complex leads to neurite expansion. Along with these changes, the formation of the NGF/ERK/CREB complex improves learning and memory function. In contrast to NGF, BDNF binds to type B Trk. This attachment recruits the PLC/PKC increases synaptic activity and plasticity. In this molecular pathway, BDNF could inhibit the BIM and reduce apoptotic response in the neurons. Further, the regulation of the PI3K/Akt/mTOR axis regulates protein synthesis and PSD-95 trafficking into the synapses. The BDNF/TrkB improves neuronal survival through Erk5/Mef pathway. The neurite outgrowth is controlled via BDNF/TrkB/JAK/STAT pathway

Neurotrophin or NGF family is the foremost investigated NTFs in neural functions [15]. Neurotrophin includes NGF, BDNF, NTF3, NTF4/5, and NT6. These factors bind to surface tyrosine kinase receptors (TrkA/B) and Neurotrophin Receptor P75 (p75^NTR^) which are highly expressed in specific regions such as the hippocampus and neocortex [16, 17]. It has shown that the p75^NTR^ mediates and adjusts neuronal migration, differentiation, and axonal projection in the embryonic and adult periods. This receptor guides the migration of NSCs from the SVZ toward the olfactory bulb. Moreover, p75^NTR^ regulates hippocampal neurogenesis and promotes behavioral performance [18–21]. TrkA/B kinases have a fundamental role in the developing and adult periods and induce neurogenesis, neuronal survival, and functional behavior upon binding to neurotrophins including NGF and BDNF (Fig. 3) [8, 16, 22, 23]. Three different transduction pathways such as MAPK/ERK/CREB, PI3K/Akt/GSK-3β, and PLCγ/PKC are regulated after the attachment of NGF to the TrkA receptor [24, 25]. The effectors can regulate cholinergic differentiation, neurite expansion, and memory enhancement. Of note, BDNF is the vastly distributed neurotrophin in the CNS and correlates with neuroplasticity, neurogenesis, and antioxidant activity in NSCs [26, 27]. This factor exerts its pleiotropic neuroprotective effects through initiating several signaling cascades. For instance, the synaptic efficiency, outgrowth neurite formation, and cell viability are associated with the modulation of PLC/PKC, PI3K/Akt, and JAK/STAT axes after the attachment of BDNF to TrkB receptor (Fig. 3) [28, 29].

The Neuropoietin family are also known as Neurokine, consists of CNTF, IL-6, IL-11, IL-27, LIF, oncostatin-M, NPN, and CTF1 [30]. These NTFs are involved in both neuronal and glial differentiation, modulation of neurotransmitter phenotype, and the survival of motor neurons via their transmembrane glycoprotein receptor. Among these factors, CNTF is produced by astrocytes to support neuronal survival and oligodendrocytes differentiation and maturation [30]. The CNTF is a protective and therapeutic factor in demyelinating diseases via the modulation of MAPK and STAT3 signaling pathways [31, 32].

**Fig. 4:**
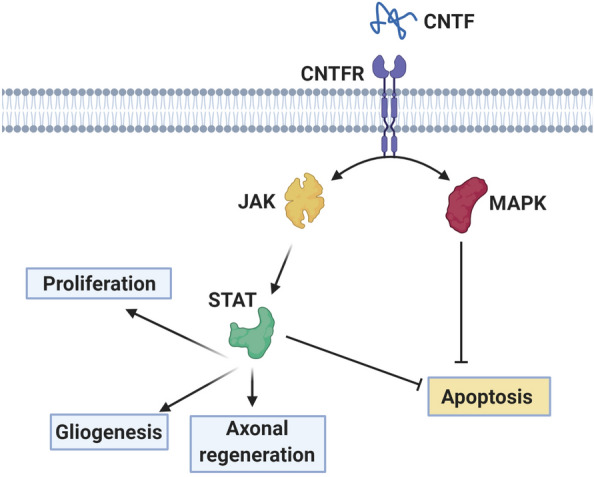
CNTF signaling pathway. CNTF/CNTFR induces the MAPK and JAK/STAT pathways to inhibit cell death. JAK/STAT pathway is recruited via CNTF to induce proliferation, gliogenesis, and axonal regeneration

**Fig. 5:**
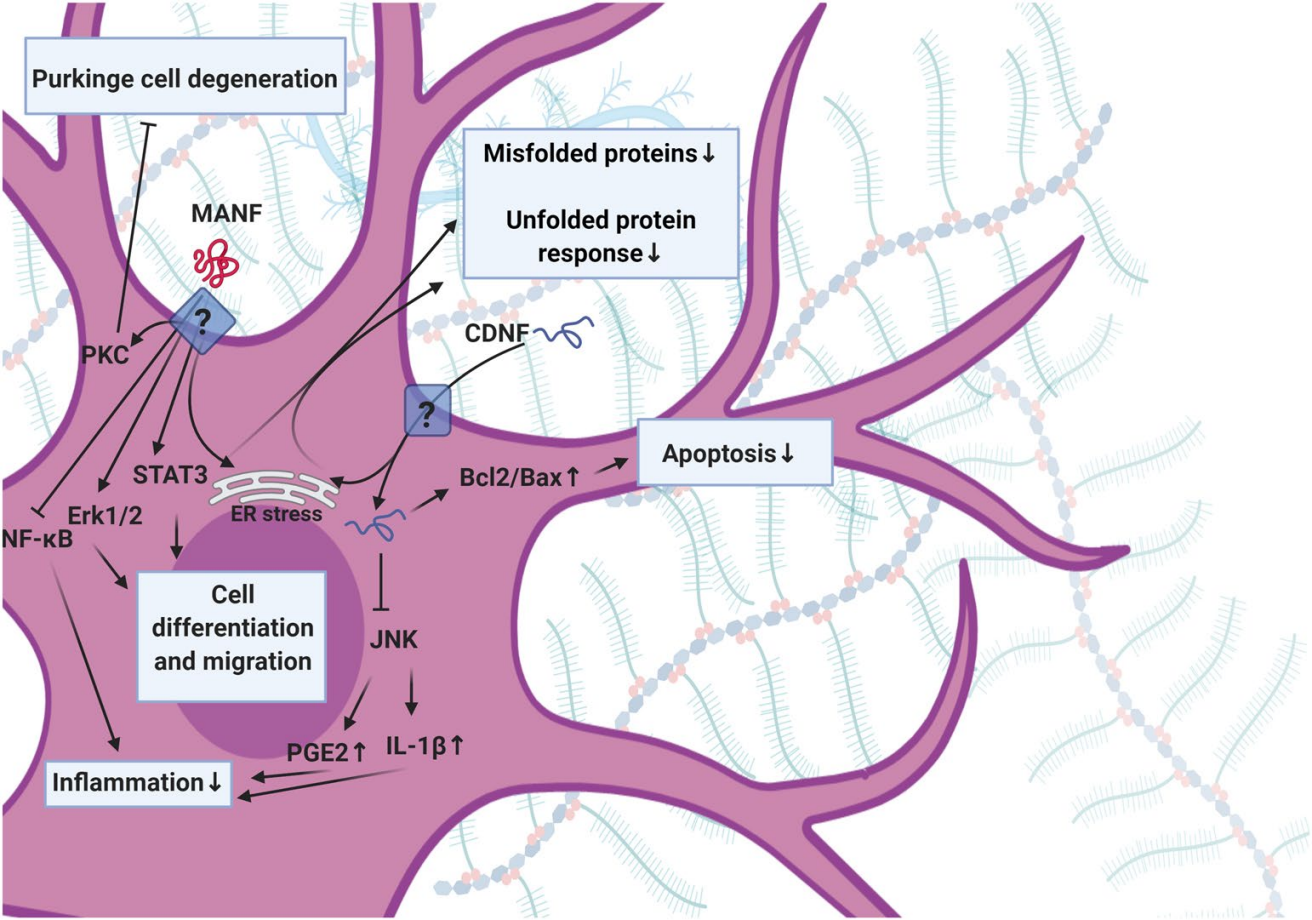
MANF and CDNF signaling pathways. Both MANF and CDNF regulate cell activity during ER stress and UPR function. MANF initiates PKC signaling and impedes degeneration of Purkinje cells. The MANF/STAT3 and MANF/ERK1/2 adjust the migration and differentiation of cells. The NF-κB signaling is regulated via MANF to control the inflammation. CDNF inhibits JNK in reducing inflammation via downregulation of the PGE2 and IL-1β. Further, CDNF increases the Bcl-2/Bax ratio and reduces apoptosis

Along with these factors, other cytokines such as CDNF and MANF can be found in the context of CNS. These factors vary in structure, sequence, and mode of action compared to the other NTFs (Fig. 4) [33]. It was suggested that CDNF and MANF are relatively stable proteins and distributed in the brain parenchyma. The CDNF and MANF are touted to control intracellular survival pathways and are interestingly effective on damaged dopaminergic, cortical, and Purkinje neurons via the regulation of ER function [33, 34]. The MANF activates PKC signaling pathway to inhibiting the degeneration of Purkinje cells [35]. Besides, the differentiation and migration of NSCs are stimulated through STAT3 and ERK1/2 signaling pathways [36]. MANF can also inhibit the p65-transcriptional activity and the expression of NF-κB-mediated target genes [37]. CDNF impedes the phosphorylation of JNK under inflammatory conditions. Further, the promotion of the CDNF/JNK axis decreases the secretion of PGE2 and IL-1β cytokines [38, 39]. The increase of the Bcl-2/Bax ratio and reduction of Caspase-3 activity can diminish apoptosis in neurons (Fig. 5).

## Preclinical vector systems for NTF delivery

To date, NTFs have been delivered via various naïve cells or engineered stem cells to overexpress one or a group of NTFs in different animal models. Besides the stem cells, the different viral vectors or several biomaterials both natural and synthetic were utilized to serve the NTFs to the neural tissues. This review will highlight available delivery systems and their possible challenges regarding NTFs into the CNS.

### Cell/stem cell-mediated NTF delivery to the target sites

The progress in targeted NTFs delivery is an important step in the regenerative medicine of CNS. During the last years, in most therapeutic approaches, different cells/stem cells have been utilized to deliver NTFs to the injury sites [40]. Stem cells are defined as distinct cells with self-renewing and differentiating abilities [40]. It is worth mentioning that cell/stem cell-based NTF delivery has dual potential efficient therapy while replacing the damaged cells, they could improve neuronal survival and axonal growth via secreting NTFs [41]. Therefore, cell/stem cell-based therapies can be the ideal vector for the long-term and site-specific NTFs production, if releasing cells or progenitors with a controllable lifetime will be engineered.

### MSC-mediated NTF delivery

MSCs are described as adherent, fibroblast-like cells with prominent proliferation capacity [42]. These multipotent progenitors can differentiate into adipocytes, smooth muscle cells, osteocytes, chondrocytes, and neuronal lineage. As a consequence, they have been used vastly in the regeneration of different injuries [43, 44]. MSCs could be isolated from various birth-related tissues such as the umbilical cord, Wharton’s jelly, and placenta as well as several adult tissues including adipose tissue and bone marrow [45, 46]. These sources particularly the birth-related tissues are the most promising tissues for optimal, easy, and non-invasive sources for harvesting the MSCs [47]. Therefore, these features make the MSCs an ideal cell source for autologous and allogeneic transplantation [47]. The MSCs can be utilized for NTF delivery, either by their innate NTF producing and secreting ability or after induction to overexpress distinct NTF [48]. In a study, the application of human MSCs in the TBI of a rat model upregulated NGF, BDNF, NT-3 coincided with the reduction of neuronal apoptosis through the phosphorylation of Akt and suppression of Caspase-3 [43]. Of note, the IV injection of AD-MSCs in ALS mice delayed motor function decline after 4–6 weeks post-transplantation. On day 100, the number of surviving motor neurons was increased in the lumbar region following the up-regulation of GDNF and bFGF in spinal cord parenchyma. In contrast to these findings, in vitro cultured AD-MSCs secreted bFGF but not GDNF. One reason would be that these contradictory data can be related to stimulatory effect of AD-MSCs on astrocytes to secret the GDNF in in vivo conditions [49]. Further data have revealed that AD-MSC-derived BDNF increased the number of TH positive cells and recovered motor function in PD rats. Moreover, TH and PCNA positive cells were ipsilaterally repopulated following the injection of AD-MSCs expressing BDNF [50].

### Genetically engineered MSCs for NTF overexpression

A plethora of studies have indicated that the direct injection of NTF resulted in loss and inactivation of NTFs by the time [51, 52]. In some pathological conditions, this phenomenon inhibits axonal regeneration and neuroma formation [52]. It is well documented that the naïve MSCs produce the trophic factors at basal levels which is closely associated with their sources [53]. Therefore, the engineered MSCs could overcome the mentioned obstacles and enhance the regenerative mechanisms leading to positive functional outcomes [54, 55]. Manipulated MSCs are capable to express specific NTF for manifold regenerative outcomes compared to the naïve MSCs. In this regard, the MSCs can be transferred with viral vectors expressing certain NTF genes. These engineered MSCs are capable to overexpress NTFs in an abundant manner [56]. A study described that the MSCs expressing GDNF increased the outgrowth of dopaminergic terminals 4 days before PD induction in rats [57]. It has been declared that MSCs expressing NT-3 advanced motor function, axonal regrowth, and neuronal survival in an SCI rat model [58]. Data support the critical role of CNTF on the dynamic growth and viability of cells and the extension of axons in mature regenerating neurons [59]. Studies in an SCI rat model showed that transplantation of CNTF expressing MSCs promoted the locomotor function assessed by the BBB scale [59]. In a similar study, transplantation of BDNF producing MSCs recovered the function of the diaphragm muscle in the unilateral SCI rat model [60]. BDNF expressing MSCs have potential to reduce atrophy of the striatum and increase neurogenesis rate in two HD mouse models including YAC128 and R6/2 [61]. Because of their low immunogenicity (low levels of MHC-II), MSCs can survive in the transplanted sites [46, 47, 62]. The existence of such capacities makes MSCs a safe, tolerable, and efficient biological vector for the production and release of therapeutic agents like NTFs into the target sites.

Despite these advantages, MSCs last for a short time (about 4 weeks). Neuroglia activation in the injured areas, promotion of hypoxia, anoikis, apoptotic and necrotic changes in transplanted MSCs increase the loss of transplanted cells [63]. To be specific, transplanted cell death activates microglia infiltration and astrocyte accumulation in the periphery of graft sites [64, 65]. It should not be neglected that MSCs can, in part but not completely, diminish the activity of immune cells inside brain parenchyma. However, the promotion of immune cell activity and recruitment overcome MSC therapeutic effects over time. Interestingly, different subsets of MSCs possess varied immunomodulatory capacities. For instance, it has been shown that bone marrow-derived MSCs present minimum proliferation capacity and maximum immune-modulatory activity in comparison with Wharton’s jelly and AD-MSCs [66]. Another issue correlated with the transplantation of MSCs is the possibility of anaplastic changes inside the brain parenchyma due to immunomodulation properties [67]. Only local injection of MSCs can introduce an appropriate cell number into the injured sites. MSCs can barely cross the BBB when injected via the IV route [68–70]. Commensurate with these descriptions, the application of novel engineering approaches is mandatory to circumvent these limitations.

### NTF-SC mediated NTF delivery

Based on a plethora of investigations, it has been shown that NTF-SCs can produce and release multiple NTFs including BDNF, NGF, and GDNF in significant amounts. Therefore, these cells can be utilized as a vehicle for delivering and serving NTFs to both CNS and PNS [56]. NTF-SCs have the potential to sufficiently migrate toward the lesion sites after transplantation in HD rats [71]. These astrocyte-like cells secrete arrays of cytokines such as GDNF, NGF, BDNF which could decrease the lesion of DA neurons and improve motor and behavioral function of PD rats [72]. Molecular investigations have revealed that NTF-SCs upregulate dopamine and regenerate the network of dopaminergic nerve end via the migration into the injured striatum [41]. Likewise, injection of NTF-SCs in EAE mice (an MS model) was considerably neuroprotective to delay animal death [73]. Histological examination indicated that NTF-SCs increased myelin sheath thickness around injured axons in the SCI rat model. Besides, the diameter of axons and the number of mature oligodendrocytes increased in the lesion areas [74]. Noteworthy, simultaneous application of NTF-SCs with other stem cell types could be a therapeutic strategy to increase the regeneration capacity. For example, it has been indicated that co-transplantation of NTF-SCs and human AD-MSCs improved motor function and MBP and increased the number of oligodendrocyte progenitors (Olig2^+^ cells) in the MS rats [75]. Regarding immunomodulatory properties, NTF-SCs can reduce gliogenesis and boost neural differentiation when co-cultured with AD-MSCs. This process leads to the orientation of NSCs toward mature neurons and improved neurogenesis. It is postulated that NTF-SCs may secret other trophic factors that have not been evaluated yet [76]. The activation of the antioxidant system is another neuroprotective mechanism by which NTF-SCs can diminish CNS injury [73]. Unlike MSCs, the NTF-SCs survive better inside the brain parenchymal, but the underlying mechanisms have not been completely understood. One reason would be that NTF-SCs display a pro-neural phenotype and this feature does not provoke immune cells to the periphery of injection sites after transplantation of NTF-SCs. Pathological examination have revealed the lack of tumor formation or adverse effect following NTF-SCs transplantation [48]. The supernatant of NTF-SCs was more protective against the quinolinic acid toxicity than that of MSCs-derived supernatant [56]. Studies point to the fact that human NTF-SCs are more neuroprotective in comparison with rodent NTFs, showing species-specific activity. For example, human NTF-SCs can secret BDNF and GDNF more than 1.5 months in in vivo conditions [56]. Along with these descriptions, NTF-SCs and MSCs can be used as cellular vehicles for the transfer of certain factors into the brain parenchyma in a autologous and allogenic manner [56, 77]. Consequently, the reduction of immune rejection can be a solution for ethical concern of stem cell vectors [77]. Successful preclinical researches have shown that NTF-SCs perform an excellent activity to mitigate the neurodegenerative. The application of NTF-SCs seems to be an effective and promising cell for treatment of neurological and neurodegenerative disorders.

### NSC-mediated NTF delivery

NSCs are characterized as multipotent and self-renewing cells with the ability to differentiate into mature neurons and neuroglia cells [78]. There are two distinct niches in the adult brain where NSCs accommodate as follows; the SVZ, and the SGZ. Under the normal conditions, the neuroblasts could migrate from the SVZ and SGZ toward the olfactory bulb and hippocampus where they commit into functional neurons, playing a key role in the smell sense and memory function, respectively [78]. According to numerous experiments, NSCs are isolated from adult or fetal/embryonic nervous tissues or even can be derived from iPSC [79]. Several trials have shown that NSCs are potent and efficient cells to replace injured cells and used as a vehicle for the delivery of growth factor [78]. NSCs can deliver NTFs to the injured sites through their intrinsic NTF releasing ability or can be manipulated to overexpress distinct NTFs. These engineered NSCs can appropriately overexpress single or several NTFs in significant levels for prolonged time. Lee et al. demonstrated that transplantation of human NSCs in AD mice up-regulated BDNF, NGF, NTF3, NTF4, GDNF, VEGF, and FGF2 levels. The implanted NSCs recovered the special memory function, ameliorated gliosis, and decreased tau hyper phosphorylation and amyloid levels via the modulation of Akt/GSK3β signaling pathway [80]. In a stroke model mouse, transplanted human NSCs can migrate to the ischemic sites and decrease significantly infarct volume, leading to behavioral function improvement within the early 24 h. Histological examination and molecular investigations have shown that human NSCs secreted BDNF and diminished activity of microglia, expression of pro-inflammatory factors, and reconstructed BBB lesion [81]. In addition to these advantages, NSCs can be engineered to secret specific NTFs. Lee et al. transplanted BDNF overexpressing NSCs in ICH mice. They found that local increase of BDNF improved angiogenesis and recovered behavioral function. Further studies have indicated that human NSCs could successfully differentiate and survive in grafted sites [82]. The other study observed that grafted NSCs-BDNF into the brain of TBI rats could progress motor function. In compression to the naïve NSCs, NSCs expressing BDNF exhibited more viability and promote neurite expansion and synaptogenesis via the expression of synaptic proteins [83]. These cells can increase the expression of the TrkB gene and phosphorylate TrkB proteins in the injured area. Further, overexpressed BDNF enhances Ras, pErk1/2, and PSD-95 in NSCs-BDNF grafted rats. It is thought that BDNF can induce Nrf2/Trx pathway [84]. Application of GDNF producing NSCs in stroke model rats triggered neurogenesis and Erk1/2 phosphorylation while the expression of MKP-1 was reduced [85]. In another study, transplantation of GDNF-tagged human NSCs into the spinal cord of athymic node rats increased differentiation into astrocyte-like cells, and transplanted cells survived for a long time (7.5 months) without a significant proliferation rate [86]. Commensurate with these descriptions, NSCs can be an appropriate source if NTF production and delivery is continued [86]. Human NSCs expressing IGF-1 generated GABAergic neurons about 10 weeks after transplantation in the AD mice. IGF-1 secretion did not change the cellular proliferation and migration functions but altered the number of differentiated cells [87]. These features demonstrate that allogenic NSCs can be successfully manipulated to produce certain growth factors under specific pathological conditions. Implantation of NSCs-GDNF into the hippocampus of mice preserved GDNF expression ability even after the differentiation into the end-stage lineages [88]. To achieve more efficient NSCs with higher regenerative capacity, these cells can be modified to secret multiple NTFs. Under these circumstances, the apoptotic and injured cells can be repopulated in the shortest possible time, and differentiating cells can integrate into the circuits of the brain to reconstruct the synapses [89]. Considering the existence of different sources to harvest the NSCs, the obtained cells may vary in NTF secretion and migration abilities [90]. One of the disadvantages of NSC transplantation is the risk of tumor formation like glioblastoma and the alloreactive immune responses in the patients who received allogenic NSCs [91]. Whether transplanted NSCs can show anaplastic changes or increase the risk of brain tumors via alteration of glial cell activity is the subject of debate.

### Immune cell-mediated NTF delivery

Several studies suggested that immune cells are capable to be used as therapeutic bio-shuttles to deliver distinct molecules into the target sites [92]. Among the immune cell subsets, macrophages are the most appropriate target cells because they are activated soon after the initiation of the inflammatory response [92]. According to the previous studies, microglia/macrophages activate and migrate to the lesion sites within 24 h to scavenge the damaged cell, debris, and secret large amounts of GDNF and BDNF [93]. These cells are critical for regulating the tissue repair stages [92]. In this regard, monocyte-macrophage lineage could provide an efficient cellular system to deliver GDNF and other NTFs to the site of the lesion within the CNS [90]. In support of this claim, Biju et al. previously utilized bone marrow-derived macrophages as a GDNF delivery vehicle [94]. Upon the expression of the GDNF, the open field activity was significantly improved in dopaminergic neuro-degenerated mice [94]. Axonal regeneration and preservation of TH^+^ neurons were investigated in both striatum and *substantia nigra* regions [94]. GDNF-expressing macrophages could successfully cross across the BBB and deliver their payload into the neuro-degenerated DA neurons following systemic administration [95]. Another macrophage-based NTF therapy was associated with the delivery of NTN to the dopaminergic lesion. Data showed that NTN secreting cells ameliorated the reduction of the TH^+^ neurons, synaptic loss in the striatum, and behavior deficiency [96]. Systemic administrated GDNF-macrophages improved almost all motor dysfunctions in transgenic Parkin Q311X (A) mice in the late stage of PD [97]. Furthermore, inflammation, reduction of DA neurons, accumulation of α-synuclein in the midbrain were all restored following GDNF delivery [97]. Chen et al. have revealed that non-toxic HSCT-based macrophage-mediated GDNF delivery improved both motor and non-motor symptoms in the MitoPark mouse model via preserving TH positive neurons [90]. This method continuously delivered GDNF to the PD-induced vulnerable DA neurons, leading to the accumulation of GDNF levels in the midbrain [90]. Indeed, inflammatory conditions such as degenerative, traumatic, and ischemic diseases lead the infiltration of the immune cells into the lesion sites coincided with the accumulation of macrophages. Therefore, application of macrophages as a NTF bio-carrier can provide more powerful therapeutic approach [95, 98]. These results showed that microglia, as one of the cells related to macrophage class, could be utilized as a natural, individualized and immune tolerable delivering vehicle for protein delivery like NTFs. Of note, macrophages can deliver several payloads without requiring special receptors [42]. Due to the use of the patient’s own cells, the risk of immune system activation is reduced or eliminated as possible [42].

## Challenges related to cell/stem cell-based NTF delivery

As described above, stem cell and cell vectors have inherent advantages and disadvantages. The stem cells can migrate and affect a wide area of the injured sites. Stem cell-mediated NTF delivery can increase regeneration of CNS via the stimulation of endogenous and exogenous cell replacement mechanisms. In response to these conditions, endogenous stem cells can proliferate, differentiate and survive through the secretion of several NTFs in the injured area after the releasing of exogenous NTFs. Simultaneously, the exogenous stem cells can differentiate in situ and replace the damaged cells [99]. Molecular investigations have revealed that stem cells can be manipulated to overexpress one or several NTFs for more efficient factor therapy. Attempts to use stem cells as NTF delivery carriers showed that stem cells can temporarily (for 6 weeks) secret NTFs after transplantation. To increase the efficiency of cell therapy, researchers have suggested repeated cell injections. This can emerge new challenges like the source for the cell because if the cells are isolated from the patient’s own body, multiple isolations and supply of required cell numbers are challenging [48]. Besides, the cryo-preservations of the cells have their challenges. For instance, freezing and thawing processes create molecular and physical damages and can reduce the capacity of protein production [48]. It should be considered that transplanted stem cells or non-stem cells may affect by the pathology and intensity of neurodegenerative diseases. Stem cells can appropriately respond to chemotactic gradients and efficiently migrate to the damaged regions [100, 101]. Indeed, this property has dual aspects. It has been indicated that transplanted cells can migrate broadly to both injured and healthy areas after transplantation. The migration of the transplant cells to the healthy areas of brain tissue other than injured sites should be controlled to preserve the optimal cell number in the injury sites [102]. Likewise, the differentiation of stem cells into undesired cell types and the possibility of tumor formation should not be neglected. Due to the possibility of immune system responses and inflammation risk, reliable and safe source of stem cells is suggested [1, 40, 101]. The prolonged immune response can generate glial scar formation around the transplanted stem cells which in turn can reduce their secreting abilities and life span [99]. Taken together, the need for invasive surgeries to administrate cells, the existence of host rejection risk, tumor formation, and ethical complications are issues that restrict the widespread use of stem cells despite numerous advantages. The applying stem cells/cells for NTF therapy of the CNS could have preponderance still, it is needed more researches to meet all positive and negative criteria before becoming a routine delivering method in clinics.

## Virus-mediated NTF delivery

The last decades have witnessed progress in regenerative therapies by the application of sophisticated biotechnological methods [40]. Viruses can innately deliver specific genes into the target cells [40, 103]. A virus is touted as an enclosed nucleic acid in a lipid capsule that can transport the specific RNA or DNA sequence into the nucleus of host cells.

### AdV-mediated NTF delivery

Adenoviruses are non-enveloped viruses, ranging from 90 and 100 nm, and have linear-double-stranded DNA (30–40 kb) with serotype-dependent activity. These viral particles are one of the most popular viral vectors for gene delivery [104]. According to many studies, AdVs have shown to be successful in NTF delivery into the CNS. Of note, the AdV-GDNF delivery in a TBI model rat enhanced neuronal survival and induced neuroprotection but did not recover the behavioral function [105]. The retrograde AdV-BDNF treatment in SCI rats decreases apoptotic signaling pathways in neurons and oligodendrocytes [106]. In an experiment, the injection of AdV-BDNF in the chronically compressed spinal cord mouse model reduced apoptosis changes in local neurons and oligodendrocytes. Similarly, the application of retrograde AdV-BDNF proliferated oligodendrocyte progenitors and increased neurofilament expression [107]. Direct intra-amniotic delivery of AdV-GFP-BDNF diminished the neural tube defect in a rat model via accumulation of BDNF in the lesion sites. Along with these changes, the expression of Caspase-3 was decreased and Bcl2/Bax ratio was elevated, indicating suppressed apoptotic response [108]. In a recent study, the injection of GFP-tagged AdV expressing GDNF vector into the hippocampus of rats promoted the function of astrocytes and neurons [109]. Relevant to the administration route, the retrograde muscular or peripheral nerve AdV injection can be repeated several times to attain acceptable outcomes [107]. The AdV vectors can be isolated from various species and easily infect a large number of target cells while carrying 8–36 kb genetic elements [110]. One of the advantages of AdV-based vectors is the expression of transgene without integration into the genome of infected cells. Consequently, the risk of tumor formation is also low. The major drawback of AdV administration is rapid activation of inflammatory response in a dose-depended manner which decreases sufficient gene expression [111]. According to above studies, AdVs can be efficient in gene transduction and will be more optimum vectors by passing time and more experimental studies.

### AAV-mediated NTF delivery

AAVs are small (~ 25 nm), safe, non-enveloped, and single-stranded DNA (4.7-kb) viruses which are belonged to the genus Dependoparvovirus, a subset of the Parvoviridae family [112]. The AAVs can be isolated from several tissues of both human and non-human vertebrates. Inside the body, AAVs are carried through the intracellular cytoskeletal network to deliver the single-strand genome to the nucleus. These viruses can be concentrated inside the CNS parenchyma. Among various serotypes, the AAV9 and AAVrh.10 can cross the BBB and infect both neurons and glial cells [113]. The delivery of AAV2/1-human IGF-1 in the SMA mouse model decreased apoptosis in motor neurons whereas the survived neurons were without function. Importantly, the treatment hindered motor function and emerged atypical muscle fiber and neuromuscular junctions [114]. Xue et al. found that EPO secreting AAV9 preserved DA neurons and improved motor function in the PD rats [115]. The combined application of an mTOR inhibitor, rapamycin, with AAV2 expressing GDNF into the rat brain resulted in abundant GDNF expression in the striatum [116]. Evidence points that application of self-complementary AAV expressing BDNF and NT-3 vectors in SCI rats regenerated injured axons, aggravated motor function, and promoted spasticity symptoms [117]. A more recent study showed that the administration of AAV7-hMANF in rats with distal middle cerebral artery occlusion enhanced the amount of the macrophages in the peri-infarct regions for short-term and improved behavioral function [118]. The following properties can be considered as the merits of the AAV as a vehicle, transduce non-dividing cells including neurons, long-time transgene expression with a single injection (about 2 years in primates), being safe and low-pathogenic and non-insertional mutagenesis [115, 119]. Of note, FDA previously accepted AAVs as suitable vectors for several clinical trials [120]. So far, the three AAV1, AAV2, and AAV6 have been approved to be utilized as a vector in clinics [121]. However, the need for large-scale vector production, high production price, AAV particle purification, possible immune system response are drawbacks associated with AAV application [113]. The apparent immune response can reduce the expression of the transgene after transplantation into the injured sites [122]. Besides, the application of higher doses can be toxic and exert inevitable side effects [115]. Molecular investigations have shown that the transduced AAV genome disappears constantly because of not-replicating episomes [113]. The major drawback of these three AAVs (AAV1, AAV2 and AAV6) is that they are not completely successful in targeted delivery of the transgene to the particular tissues or cell types. According to data from Hsu et al. study, the new identified human AAV, namely AAVv66, can pave the road for viral vector gene delivery. AAVv66 spread superiorly when injected in hippocampus, the most critical brain region in neurodegenerative disorders like AD [121]. These data showed that AAV-based delivery is relatively efficient to deliver NTF gene during neuro-related disorders.

### Lentivirus (LV)-mediated NTF delivery

The virus-based vectors are affording effective long-term gene delivery [123]. The LV-based gene delivery vectors have been effectively transferred the target genes into the various sites within the CNS [123]. The LV is a member of the *Retroviridae* family with 100 nm in diameter. This single-stranded RNA genome (∼ 9.7 kb) viruses can penetrate the envelope of the nucleus and infect both mitotic and non-mitotic cells like glia and neurons, respectively [112]. The lentiviral vectors have been immensely purified and used as safe vectors for therapeutic applications. Preliminary data have shown that LV-BDNF delivery in SCI supports axonal elongation up to 9 mm in neural progenitors graft-derived GABAergic and glutamatergic neurons [124]. Further, LV-BDNF or -NT-3 delivery from a multichannel PLG bridge regenerated and remyelinated axons in the SCI model of rats [125]. Combined administration of LV-NT-3 and short hairpin (sh) RNA for NG2 (one of the main inhibitory chondroitin sulfate proteoglycans) in an SCI rat model increased neurons, NG2 levels, and locomotor function. Importantly, administration of LV-NT-3 plus LV-shNG2 diminished the astrocyte levels and the size of the scar [126]. Thomas et al. delivered LV-NT3, LV-SHH, and/or LV-NT3-SHH from the PLG bridges into the spinal of SCI mouse model [127]. Axonal regeneration and re-myelination were notified. These changed were along with the increase of Olig2^+^ and GFAP^+^ cells [127]. Interestingly, in situ increase of NT3 improved axonal myelination through the promotion of oligodendrocyte and Schwan cell activity whereas SHH only promoted oligodendrocyte bioactivity [127]. The delivery of LV expressing GDNF to AD mice models enhanced learning and memory function while simultaneously reduced cognition capacity [128]. In another experiment, the introduction of LV-GDNF in AD animals increased BDNF levels but the level of amyloid and tau was significantly unchanged [128]. Despite these advantages, oncogenic mutation can occur in some cases following LV genome integration into the host cell genome. This is touted as the main concern of safety in in vivo conditions [129]. By contrast, LV-based NTF delivery has numerous benefits like long-term transgene expression, low inflammation rate, retrograde transportation, large size gene insertion (about 9 kb), and simultaneous expression of several genes [130]. Following the diagnosis of a disorder, the NTF gene should be delivered in the proper area of the CNS to have effective results so vesicular stomatitis virus G-glycoprotein-pseudotyped LV is an applicable vector for site-specific transduction [129, 131]. Humbel et al. developed an LV-based vector that can transfer genome especially into the astrocytes and be retrogradely transported in interconnected brain circuits. This LV vector can be utilized in neurodegenerative diseases like AD that vastly damage the brain [130].

## The challenges of virus-based NTF delivery

Controlling NTF gene production and terminating its expression are some challenging features of viral vectors. To this end, researchers have tried to construct self-inactivating viral vectors for in vivo applications. This strategy has other challenges too [40]. For example, in AdV-vector infected cells, the expression of target genes is transiently [111]. Besides, these vectors promote cytotoxic effects to the host cells and provoke inflammation response [59]. However, the development of high-capacity AdVs, namely HC-AdVs, reduced the risk of cytotoxicity and immune response [110]. Moreover, evidence supported that the LV, not only, does not alter the cellular behavior but also it does not significantly affect the immune system. In addition, the physical properties of LV, even after encoding a gene, do not change [127]. Of note, the LV genome integrates into the target cell genome which has dual positive and negative aspects, constant gene expression even in daughter cells, and the raise of tumor formation risk [103, 110]. The viral vectors diffuse scanty when injecting intracranial in addition while in systemic administration about 1% of injected vectors reach into the brain. It is suggested that virus entrance and transgene delivery are associated with the collaboration of particular receptors [42]. The low particle size (25 nm), enables the AAV to diffuse easily in the injected sites [103]. The other limitation is the size of the transgene that each virus is capable to transport [103]. The AAV can carry about 4.7 kb but these values are 9 and 30–40 kb for LV and AdV vectors [103, 132]. Compared to LV-based vectors, AdV vectors seem to be not tumorigenic [133]. The viral vectors are appropriate for CNS gene delivery some novel technologies such as genome editing can be combined with viral NTF delivery which will rectify our knowledge about neurobiology and CNS-related diseases hence, will pave the road for more efficient treatments.

## Biomaterial-mediated NTF delivery

A biomaterial is described as a material with the interaction ability to the biological tissues. In addition to the protection of transplanting cells, biomaterials help these cells to revive the ECM. Although cells loss is the main concern in neurodegenerative disorders, the ECM with the fundamental protective role should be more noticed [134]. It is thought that ECM is significantly injured during CNS disorders and hence reconstruction of ECM using appropriate biomaterials with the ability to carry and release the NTF could be a useful strategy [134]. Biomaterials can be designed as a carrier of proteins and drugs. Besides, they can serve as a platform for cells proliferation and differentiation. The researches have been shown that protein-loaded biomaterials advanced the therapeutic ability to transplant cells [135]. Biomaterials can be locally engrafted and slowly release the targeting molecules. In some disorders like stroke, biomaterials especially the hydrogels can be loaded in the cavities and functionally contribute to cell expansion [134]. Biomaterials serve local and site-specific delivering approaches [136]. Based on many types of research, various natural and synthetic biomaterial-drug and protein delivery systems have been developed.

### Natural biomaterial-based NTF delivery

Natural biomaterials can be derived from human, animals, and plants tissues [137]. The natural biomaterials are classified into protein and polysaccharide scaffolds. Protein-based natural biomaterials consist of collagen, gelatin, keratin, fibrin, etc. This group of biomaterials can be generally derived from human and animal tissues. The polysaccharide-based natural biomaterials include hyaluronan, alginate, cellulose, chitin, etc. The polysaccharide biomaterials are commonly obtained from agar, alginate, and in some cases from microbial sources [138]. Natural biomaterials possess a low toxicity rate, biocompatibility, biodegradability, and remodeling advantages [139]. The natural entity of natural biomaterials enhances the risk of immune responses. From a physicochemical viewpoint, these biomaterials are thermally and mechanically resistant. Chitosan is a well-known chitin derivative that consists of β (1–4) linked glucosamine and N-acetylglucosamine [140]. The near structure of chitosan and glycosaminoglycans make it a safe, non-toxic, and degradable natural-based hydrogel [139]. Intranasal delivery of NGF-loaded chitosan enhanced almost 14-fold the bioavailability of NGF at the target sites [141]. Another natural polysaccharide is hyaluronan with a linear structure that can be found in neural ECM. Studies have shown that hyaluronan is one of the widely applied injectable biomaterials to deliver therapeutic agents [142]. Hyaluronan is biocompatible and non-immunogenic biomaterial [140]. The injection of hyaluronan-based hydrogel enriched with BDNF improved motor function in stroke mice due to axonal regeneration in cortical and cortico-striatal systems [143]. Along with these changes, immature neurons migrated successfully and survived in the peri-infarct cortex [143]. The hyaluronan-BDNF could diffuse over the infarct site three weeks after transplantation [143]. Methylcellulose is a water-soluble polymer that is derived from Cellulose [139]. Methylcellulose can combine with hyaluronan to form HAMC hydrogel. This hydrogel possesses several merits such as injection capacity, biocompatibility, fast-gelling rate, and degradability, and controlled releasing capacity of target molecules into the injured sites [144, 145]. The HAMC is bioresorbable and can degrade in about 3–7 days following CNS injection [146]. The transplantation of HAMC hydrogel harboring EPO enhanced the proliferation and maturation of neural cells and mediated inflammation. HAMC hydrogel-EPO decreased the size of the stroke cavity and apoptosis in the lesion site of cortex and SVZ in the stroke model mice [147]. Another study indicated that intrathecal administration of HAMC hydrogel enriched with NT-3 released the NT-3 for 28 days in SCI rats [148]. The persistence of NT-3 in the target sites regenerated and enlarged the axons without induction of the astroglial response [148]. In a more recent study, transplantation of HAMC hydrogel enriched with KAFAK and BDNF into the SCI rat model improved neurological function and neuronal survival. This method also appreciably decreased the expression of pro-inflammatory cytokines, the formation of the glial scar, and the cyst cavity [149].

Gelatin, a natural polymer, is biocompatible, biodegradable with little immune response activity in in vivo conditions [150]. It has been reported that intranasal delivery of phospholipid-based gelatin nanoparticles supplemented with bFGF in the PD rats yielded some neuroprotective effects such as improved DA function in synapses and the PD rotational behavior [150]. The gelatin nanoparticles-bFGF increased local olfactory bulb bFGF when administrated intranasally in comparison with direct bFGF injection [150]. Like gelatin, the natural adhesive property makes the collagen a useful hydrogel for cell delivery to the target sites [64, 151]. The application of type I collagen hydrogel containing GDNF-expressing MSCs significantly moderated neuroglia activation in the striatum [64]. The simultaneous use of collagen can increase the viability of transplanted MSCs and the local GDNF level [64]. Similar findings have confirmed that the delivery of collagen conduits harboring NT-3 into the spinal cord in the rat model not only improved axonal extension but also increased the half-life of NT-3 up to 4 weeks [152]. Like gelatin and collagen, other natural biomaterials have been used in in vivo conditions. For instance, fibrin can be an autologous scaffold for NTF delivery into the target sites. Indeed, fibrin has low immune response capacity and cytotoxicity [139]. A fibrin scaffold with a heparin-based delivery system was used for the controlled release of NT-3 in the SCI model of rats [153]. This strategy promoted neuronal fiber density and reduced glial scar formation [153]. In a similar study, the combined injection of fibrin scaffold-NT-3 and PDGF with ESNPC into the SCI rat model increased the survival of ESNPC and the number of ESNPC-derived mature NeuN^+^ neurons in the lesion site [154]. The above studies have shown that natural-based biomaterials offer targeted and efficient NTF delivery into the CNS.

### Synthetic biomaterial-based NTF delivery

Synthetic biomaterials serve controlled degradation and have a more favorable mechanical and thermal resistance [140]. Synthetic biomaterials lack immune response capacity and can produce on large scales [140]. One of the drawbacks of synthetic biomaterials is that they are not enough biocompatible. PNIPAAm is a synthetic hydrogel with thermal sensitivity [139]. Besides, PEG is a widely used hydrated and nonionic hydrogel in in vivo conditions [93]. It has been shown that PNIPAAm-g-PEG-BDNF and PNIPAAm-g-MC-BDNF treatment improved axonal regeneration in the SCI rats [155]. Noteworthy, inflammatory responses against the hydrogels were trivial and tolerable in these rats [155]. Amphiphilic DCH is biocompatible and biodegradable (in about 8 weeks) synthetic hydrogels with suitable integration ability to CNS tissue. The injection of DCH did not result in toxicity or unfavorable inflammation. Delivery of DCH-NGF into the mouse forebrain released the NGF for about 4 weeks and caused hypertrophy of cholinergic neurons [136]. PGA diblock copolymer is a safe and biocompatible scaffold. Utilization of nano-particle polyion complex with PEG/PGA-BDNF in ischemic stroke mice model improved memory and cognitive function and ameliorated depression [156]. PLGA is one of the vastly used biomaterials for drug delivery. PLGA is a biocompatible component when implant into the CNS [93]. Khalin and co-workers found that injection of poloxamer 188 (PX) coated PLGA nanoparticles-BDNF in TBI mice restored cognition [157]. In a more recent study, it was suggested that PLGA/GO electrospun nanofibers-IGF-1 or BDNF improved locomotor function, increased the number of neurons in the lesion site, and moderated the formation of the cavity [27].

## The challenges of biomaterial-based NTF delivery

The researches have shown that biomaterial can be administrated via systemic routes and locally into the CNS. The local delivery reduces the loaded amount of target molecules and prevents the unwanted effects of target molecules in other organs. In general, the application of biomaterials needs low-invasive manipulation with large-amount delivery of target molecules into the injured sites [135]. In the selection of delivery routes, some properties such as degradability, safety and non-toxicity, and adjustability to release the agent should be considered. As a common role, the biomaterials used for CNS regeneration should be injectable. It should be remembered natural biomaterials can be immunogenic but are not toxic. By contrast, synthetic components do not create inflammation but can trigger cytotoxicity. For efficient regeneration and effective NTFs delivery, the biomaterials should degrade slowly [134]. The releasing rate of NTF through the biomaterials is to be calculated in in vitro and in vivo conditions [158]. Various shapes of synthetic biomaterials can be developed while this capacity is restricted in natural-based scaffolds. In addition, the natural biomaterials are not thermal resistant and caution should be taken in the procedure of fabrication [137]. Despite these advantages and disadvantages, data have demonstrated that both natural and synthetic biomaterials did not provide desired results when applied alone and their combinations seem to be more efficient [139]. The combined biomaterials are biocompatible with suitable mechanical properties and thermal strength [137]. The field of biomaterial-based NTF delivery has a developable and improvable road and needs more researches.

## Preclinical routes for the NTF vectors delivery

Various methods have been utilized for NTF delivery into the CNS. IV, intramuscular and intranasal routes can be used as indirect CNS delivery approaches [159]. In all these routes, the agents should pass the BBB to reach the CNS parenchyma. Indeed, the integrity of BBB limits the access of these factors to the injured sites [159]. However, the mechanism of the IN delivery route is not clear [160]. The intra-cerebrospinal fluid injection and local intraparenchymal administration are direct CNS delivery methods that can bypass the BBB [159, 161]. It seems that the way of administration influences CNS concentration, distribution, and ultimately possible neuroprotective properties of regenerative factors.

### IV NTF delivery

IV injection can transmit various therapeutic agents and vectors into the circulatory system. This route is mostly applied through the tail vein in rodents. In an IV administration, the injected agents immediately reach the blood circulation and can be served to different organs inside the body. This approach can be utilized for the delivery of NTF, NTF-SCs, and viral vectors. The IV injection of human MSCs 1 day after TBI in rats could show therapeutic effects. The results showed that MSCs can cross the BBB and produce neurotrophins [43].

In systemic administration of AD-MSCs, these cells migrated into the CNS, muscles, and spleen. AD-MSCs can be detected inside the gray and white matter of the spinal cord in ALS mice. The recruitment of AD-MSCs up-regulated the bFGF and GDNF levels, showing paracrine activity of AD-MSCs [49]. Like AD-MSCs, macrophages could pass the BBB and reach the lesion sites when injected systemically [94]. Besides the brain parenchyma, the IV injected macrophages could migrate into the kidneys in large amounts while in low levels are directed into the spleen, liver, and lungs. Multiple types of researches delivered GDNF or NTN secreting macrophages into the CNS via IV injection in neurodegenerative model rodents [90, 94–97]. In addition to cells, IV injection of viral vectors and certain nanoparticles have been done to deliver NTFs into the CNS parenchyma. Given the numerous advantages of AAV-based vectors for NTFs delivery, it was suggested that intravenously injected AAV9-GDNF can pass the BBB and delivered GDNF into the CNS [162]. In a study, poloxamer 188-coated PLGA-BDNF nanoparticles were used. Data showed that this system is eligible to cross the BBB and deliver BDNF into the brain in a TBI model [157].

Because of the existence of specific paracellular and transcellular pathways in BBB to regulate the delivery of biomolecules into the brain parenchyma, the efficiency of target delivery into brain tissue via the IV route is restricted [147]. Ultrasound imaging techniques can be utilized for diagnostic and therapeutic purposes to assess the function of BBB [163]. When FUS and MBs are used simultaneously, BBB is temporarily permeabilized via loosening the tight connections between the endothelial cells hence enhances paracellular entrance and transcellular transportation via caveolae-based mechanisms [164]. As a correlate, the combination of FUS and IV injection can serve as a non-invasive and controllable delivery method. The BBB recovering period is associated with acoustic pressure and the size of the bubble introduced to this barrier [83]. Previous experiments have shown that the opening and loosening of BBB via several approaches can facilitate the cross of several therapeutic substances from blood into the brain [147]. In this regard, it has been shown that the cross of BDNF from BBB increased about 20-fold when the MBs-BDNF technique was combined with MRI-guided FUS administration [165]. It seems that MRI-guided FUS administration is also useful for the delivery of GDNF plasmid DNA liposome in HD mice [166]. In addition to sufficient targeted delivery of MBs-BDNF/GDNF/NTN administration, this approach can initiate molecular signaling of pyramidal neurons in the hippocampus of mice [83].

### The challenges related to NTFs delivery via IV route

The IV injection is a usual and simple way for drug delivery with the ability to repeat injections. In addition to its less invasive nature, the IV injection has a low infection risk. However, NTFs delivered via IV routes need time to cross the BBB and reach brain parenchyma in in effective concentrations. In most circumstances, to achieve sufficient CNS doses, high levels of substances should be intravenously injected. The systemic administration of NTFs, NTF-SCs, viral vectors, NTF-loaded biomaterials can lead to uncontrolled biodistribution into the non-specific organs [167]. For example, systemic administration of NGF can cause hyperalgesia, muscle pain, and weight loss [3, 168].

### IM NTF delivery

IM injection is a common route for the delivery of viral vectors. Inside the muscle tissue, the viral vectors use retrograde transportation via axons to reach the CNS. In support of this notion, the injection of AdV-BDNF into the bilateral sternomastoid muscles transferred vectors to the injured sites via retrograde transport using spinal accessory motor neurons in SCI model rats. The AdV-BDNF could reach the spinal cord and decrease apoptotic signaling pathways in neurons and oligodendrocytes [106]. In another study, administration of AdV-BDNF into bilateral sternomastoid muscles of chronically compressed spinal cord mice led to the restoration of oligodendrocyte progenitors and neurofilament expression via the axons of the spinal accessory nerves [107].

### Challenges associated with IM delivery of NTFs

As above-mentioned, viral vectors can reach target sites through the axons of motor neurons in a retrograde manner. To reach the motor neurons, innervated muscles should be selected as the injection site. In the latter phase, viral vectors internalize into the nerve axons and reach soma and nucleus. Inside the nucleus, the gene expression is initiated which follows by the production of NTFs in the cytoplasm [169]. Like the IV route, the IM route is a repeatable and slightly invasive way for viral vector administration while in this approach the possibility of gene expression in other non-specific sites is low. According to experimental data, about 1 to 1.5 after IM injection the expression of target molecules is initiated inside the cervical spinal cord area [106]. It should not be forgotten IM injection is relatively efficient and useful for spinal-related disorders including SCI and ALS rather than brain injuries, restricting the application of this method for all CNS pathologies. The existence of immune system responses at the site of injection is another drawback that needs further attention.

### IN delivery of NTFs

Earlier studies have shown that IN delivery is a promising route for extrinsic therapeutic NTF administration into the brain [40]. The properties such as non-invasive manipulations, quick absorption rate, simple repeating dosage, and reduction of non-target biodistribution make IN delivery superior to the systemic delivery routes [40, 170]. There are three hypotheses for substance absorption through IN administration as follows; (I) Nerve pathway: the factors are carried through the axons of olfactory or trigeminal nerves [167]. Besides, the exposure of olfactory neurons dendrites into the nasal cavity can serve this facility [171]. (II) systemic circulation: in this pathway, the agent can enter blood flow and reach the brain, and (III) lymphatic pathway [167]. Of note, numerous experiments have offered that the RMS plays a fundamental role in IN drug administration [170]. Intranasally administration of chitosan-NGF hydrogel in rats boosted the bioavailability of NGF about 14-fold [141]. This approach has been applied for the delivery of BDNF, NT-4, CNTF, and EPO into the rat’s brain [172]. It is thought that during the early 25 min the brain concentration of NTFs reaches up to 0.1–1.0 nM, leading to activation of the PI3K/Akt pathway which is associated with cell viability [172]. In focal cerebral ischemic rats, IN injection of NGF reduced neurons toxicity, induced proliferation rate, and increased NeuN expressing cells in SVZ and striatum [173]. Very high levels of bFGF can be delivered into brain parenchyma via IN spray when compared to IV and IN solution delivery in AD rats, leading to improved cognition capacity [174]. This effect would be related to the evenly distribution of droplets rather than local injection. Similar to this study, IN administration of NGF into Aβ expressing TBI rats caused marked reduction of Aβ1-42 deposits and recovered the motor and behavioral function [175].

### Challenges associated with IN delivery of NTFs

According to the promising data from the animal experiments, IN administration can circumvent the BBB obstacle and deliver successfully NTFs to the brain [158]. Using this approach, it is suggested that the NTF therapy becomes applicable in patients suffering from CNS-related disorders. Although several studies were successful and their results presented strong evidence about the efficiency of IN route, the entity of some CNS disorders and impaired axonal retrograde transportation following neurodegenerative disorders account for lack of efficient delivery into the brain parenchyma. However, the IN injection is a kind of non-invasive and simple route with a low risk of infection [160]. The respiratory and olfactory epitheliums serve relatively vast areas for quick administration absorption. Unfortunately, it should be noted that the volume of IN injection is small and mucociliary clearance can reduce the CNS diffusion of target molecules [159].

### CSF delivery of NTFs

The CSF is a clear body fluid that circulates inside the ventricles, canal systems, and subarachnoid space of the brain and spinal cord [176]. The circulating CSF can reach most of the regions inside the CNS hence is a suitable fluid for agent deliveries into the CNS. To access the CSF fluid, the agent can be administrated through ICV and IT routes. In rodent models of neurological disorders, the ICV and IT routes are widely utilized for therapeutics administration. For ICV and IT injection, the agents should be delivered directly into the lateral ventricle and subarachnoid space of the brain and the spinal cord respectively [177]. The ICV transplanted NTF-SCs in MS model mice enhanced the survival period of mice [73]. Of note, the injection of human NSCs in the AD model via ICV led to the successful migration of transplanted cells to several brain regions except the hippocampus [80]. In another study, GDNF expressing NSCs were administrated via the same approach and into the ipsilateral lateral ventricle of stroke model rats, and improved neurogenesis in the marginal zone of ischemic striatum was obtained [85]. Besides the cells, the viral vectors can be injected into the lateral ventricle for NTF gene therapy. The AVV8-BDNF administration into the lateral ventricle of AD mice could upregulate the BDNF level through enhancement of BDNF gene expression [120]. Like these studies, the IT injection of HAMC hydrogel enriched with NT-3 in rat spinal cord injury model promoted ventral circulation of NT-3 and expression of this factor in the spinal cord for 4 weeks [148].

### Challenges associated with CSF delivery of NTFs

CSF administration can present the NTF to the broad regions inside the CNS. Approximately 100% of the injected agents can be delivered to the brain and spinal cord through CSF hence, the dose of an agent for ICV and IT is approximately programmable [178]. The CSF is invasive but the IT infusion is less invasive than the intraparenchymal route [179, 180]. Using the CSF route, NTF-SCs, viral vectors, and NTF-loaded biomaterials can reach directly the CNS, without circulating in the bloodstream and transferring to other non-targeted organs [181]. A low dose of protein and vectors is required in comparison with the IV route. However, the CSF administration needs technical skills and invasive surgical cannulation with a high degree of accuracy. The injection cannula should be located in correct coordination with a millimeter range margin of error. The cannula needs protection and may be stuck in a cannulated rodent for repeated ICV injection. Despite the advantages of the CSF route, the capacity of CSF for receiving the administration is restricted [177]. There is an infection risk because of the parenchymal penetration of the cannula and the slow administration in which circulation of CSF can diffuse the infection throughout the CNS [161, 182].

### Local intraparenchymal NTF delivery

As above described, the BBB is the main obstacle in the CNS delivery of therapeutics. Hence direct parenchymal delivery of therapeutics seems to be more effective. We will describe various parts of CNS tissue that are mostly utilized for NTFs, NTF-SCs, viral vectors, and NTF-load biomaterials delivery. The striatum is a common region for gray matter injection, especially in PD model rodents. Voutilainen et al. administrated single CDNF, GDNF, and a combination of CDNF and GDNF into the striatum of PD rats. They reported that DA neurons function improved following CDNF or GDNF delivery. Noteworthy, the combined CDNF and GDNF delivery caused more trophic effects [183]. Histological examination showed GDNF expressing MSCs can survive for about two weeks in the PD rats following direct administration into striatum [57]. Like this study, several studies have shown that striatal transplantation of NTF-SCs in PD and HD model rodents was effective and levels of NTFs were detectable after 4 weeks [41, 56, 71, 72]. In a study, Matlik et al. infused AAV7-hMANF into the subcortical region of stoke model rats and found that the hMANF could not decrease the injury volume but can summon the macrophages toward the lesion site [118].

Spinal cord injection is frequently evaluated in various studies in rodent models of spinal cord injuries and spinal demyelination. Different cells, viral vectors, and biomaterials were utilized to deliver the NTF to the injury site. The MSCs-NT-3 [58], MSCs-CNTF [59], NTF-SCs [74, 75], hNSCs-GDNF [86] were grafted into the spinal cord and were successful in NTF delivery and recovery of the injury. The observation revealed that the viral vectors for NTF are neuroregenerative for spinal cord injuries for example the scAVV-BDNF [117], LV-BDNF [124], LV-NT-3 or BDNF [125], and LV-NT-3 [126]. The viral vectors infusion into spinal parenchymal in these studies could reduce the injury of the spine and ameliorated the function of rodents. Collagen conduits carrying NT-3 [152] and the fibrin scaffold-NT3 delivered the NT-3 [153] in a controlled manner and regenerate the axons.

### Challenges associated with local intra-parenchymal delivery of NTFs

Studies have shown that the NTFs or their vectors can cause more efficient results when locally delivered into the special brain or spinal parenchyma. Intraparenchymal infusion causes bypassing the BBB which is the main obstacle in CNS achievement. Despite these advances, intraparenchymal administration requires invasive and high accurate surgery procedures [184]. To this end, an appropriate cannula is needed for straight injection of the agent into the brain and spinal tissues to minimize tissue irritation. The cannula can move in the injection site which can cause incorrect targeting [179]. Although the parenchyma of CNS restricts the diffusion of proteins [185], the NTFs can spread through neuronal internalization [158]. The observations have shown that the NTF-SCs can migrate through the CNS tissue and reach the injury site [64]. The direct-injected therapeutics have higher local concentration and longer half-life inside the CNS which can have dual aspects, upregulates their positive effects via presenting the agent directly to the injury site or in some cases can cause neurotoxicity [158, 185, 186]. In intraparenchymal injection, the therapeutic agent can reach deep brain regions like the striatum and hypothalamus that can be more efficient for regeneration [179]. One of the great merits of targeted parenchymal injection of therapeutics is the avoidance of CSF circulation which may deliver the therapeutic to the undesired area for example the spinal cord in the TBI model. The parenchymal injection can deliver the agent to the specific site of the brain which is important in degenerative diseases like PD. Further, the infusion can be applied in the white matter to deliver the therapeutic agent to the connective tracts hence, can be effective in spread neurodegenerative like AD [179]. The infusion rate should be very slow because the parenchyma of CNS does not have space for receiving a high amount of agents in fast injection. The injection can disintegrate the neural networks and cause local injection site damage [187]. The sterility should be highly considered because of direct parenchymal injection and there is the risk of infection. In direct parenchyma injection, almost the entire volume can be presented to the neural cells and have a high concentration in the injection site. High concentration may have dual effects, therapeutic effects, and in some cases neurotoxic effects because of high local concentration in injected parenchyma [186].

## Conclusion

Up to date, various NTF delivery vectors and systems have been applied to deliver exogenous NTFs into the CNS with promising results. The therapeutics can receive into the CNS via various routes. Of course, each of these systems and routes encompasses limitations. The superiority of stem cells in comparison with viral vectors is the lack of cytotoxic concern and transgene size limitation. On the other hand, the viral vectors have a very low risk of tumor and glial scar formation. The NTF-loaded biomaterial delivery systems seem to be successful but they need more studies. The studies have demonstrated that both biomaterials did not provide desired results when applied alone and the combination seems to be more efficient. The combined biomaterial systems consist of both natural and synthetic substances with appropriate biocompatibility and suitable mechanical properties and thermal strength [137]. Future studies should improve stem cell-based vectors to have an excellent source for stem cells with controlled or lack of immune response and consequent rejection and tumorigenic risks, and optimum time of secretion. For viral vectors, optimum packaging capacity, continued gene expression and the most important the immune response are some obstacles. Unfortunately, some in vivo delivery routes like repeated stem cell transplantation do not apply to the clinical setting. Because of cell source limitation, invasive administration route, the half-life of the vector, and the amount of releasing NTFs are probably the reasons why the application of NTFs was not efficient in the regeneration of target tissues mainly the brain for the long term. Each system has benefits and can ameliorate the symptom of CNS disorders and reduce their progression. So, it is important to continue researching on optimizing cellular, viral vectors, and biomaterial systems to providing standards before clinical applications.

## Data Availability

The datasets generated and analyzed during the current study are available from the corresponding authors on reasonable request.

## Abbreviations

AAV: Adeno-associated virus
AdV: Adenovirus
AD-MSCs: Adipose tissue-derived MSCs
AD: Alzheimer's disease
ALS: Amyotrophic lateral sclerosis
bFGF: Basic fibroblast growth factor
BBB: Basso-Beattie-Bresnahan
BDNF: Brain-derived neurotrophic factor
CTF1: Cardiotrophin1
CNS: Central nervous system
CDNF: Cerebral dopamine neurotrophic factor
CSF: Cerebrospinal fluid
CNTF: Ciliary neurotrophic factor
DCH: Diblock copolypeptide hydrogels
DA: Dopaminergic
ESCs: Embryonic stem cells
ER: Endoplasmic reticulum
EPO: Erythropoietin
ESNPCs: ESCs-derived neural progenitor cells
FUS: Focused ultrasound
GDNF: Glial cell-derived neurotrophic factor
GFAP: Glial fibrillary acidic protein
PLGA/GO: Graphene oxide (GO)-incorporated PLGA
GFP: Green fluorescent protein
HSCT: Hematopoietic stem cell transplantation
HD: Huntington’s disease
HAMC: Hyaluronan/methylcellulose
iPSC: Induced pluripotent stem cells
IL-6: Interleukin-6
ICH: Intracerebral hemorrhage
ICV: Intracerebroventricular
IM: Intramascular
IN: Intranasal
IT: Intrathecal
IV: Intravenous
LIF: Leukemia inhibitory factor
MANF: Mesencephalic astrocyte-derived neurotrophic factor
MRI: Magnetic resonance imaging
MSCs: Mesenchymal stem cells
MBs: Microbubble
MKP-1: Mitogen-activated protein kinase phosphatase-1
MBP: Myelin basic protein
NCAM: Neural cell adhesion molecule
MS: Multiple Sclerosis
NGF: Nerve growth factor
NPN: Neuropoietin
NTFs: Neurotrophic factors
NT6: Neurotrophin
NTF3: Neurotrophin-3
NTF4/5: Neurotrophin-4/5
NTN: Neurturin
Nrf2/Trx: Nuclear factor (erythroid-derived 2)-like 2/Thioredoxin
PD: Parkinson’s disease
PGA: PEG/poly (L-glutamate)
PNS: Peripheral nerves system
PLGA: Poly (lactic-co-glycolic acid)
PNIPAAm: Poly(N-isopropyl acrylamide
PLG: Poly (lactide-co-glycolide)
PCNA: Proliferating cell nuclear antigen
PGE2: Prostaglandin E_2_
PKC: Protein kinase C
RMS: Rostral migratory stream
STAT3: Signal transducer and activator of transcription 3
ERK1/2: Signal-regulated kinase 1/2
SHH: Sonic hedgehog
SCI: Spinal cord injury
SMA: Spinal muscular atrophy
SGZ: Subgranular zone
SVZ: Subventricular zone
NSCs: Neural stem cells
TBI: Traumatic brain injury
RET: Tyrosine–protein kinase receptor
TH: Yyrosine hydroxylase
